# It's time for physicians to return to psychoanalysis: the Michael Balint Scholarship for Medical Students Interested in Psychoanalytic Work

**DOI:** 10.1192/bjb.2024.96

**Published:** 2024-12

**Authors:** Himanshu Agrawal

**Affiliations:** Associate Professor and Chair of APsA's Medical Student Education Committee, Department of Psychiatry and Behavioral Medicine, Medical College of Wisconsin, Milwaukee, Wisconsin, USA. Email: hagrawal@mcw.edu.

The American Psychoanalytic Association (APsA) is an association of psychoanalysts in the USA serving as a scientific and professional organisation with a focus on education, research and membership development. Founded in 1911 by Ernest Jones (with support from Sigmund Freud), APsA is the second oldest American psychoanalytic organisation and it comprises 34 training institutes and 38 affiliate societies. APsA has over 3000 members, including psychiatrists, clinical and experimental psychologists, and social workers.^1^ Membership of APsA, from its founding in 1911 until 1989, was limited to physicians, following the belief at that time that psychoanalysis could gain acceptance in the USA only if it were presented as a treatment for a medical disorder. APsA held to this position even after clinical psychology became a recognised healthcare profession. In consequence, many members of recognised healthcare professions, particularly clinical psychologists, were excluded not only from membership of APsA but also from training in its approved institutes.^2^

In the 1980s, members of the American Psychological Association joined in a successful lawsuit against APsA, challenging these policies. In 1989, APsA, along with the International Psychoanalytical Association and the New York Psychoanalytic Society and Institute, agreed to admit non-physicians for training on the same basis as physicians.^3^

Psychoanalytic training and practice in the USA has undergone necessary major reform in recent decades to address its reputation for being dogmatic and accusations of elitism. This includes a public apology for its stance on homosexuality,^4^ increased flexibility in training criteria and a concerted effort to reconcile with underrepresented populations. Now, APsA faces a new challenge as the number of physicians interested in psychoanalysis has been steadily dwindling over the past few decades. In an attempt to address this, in 2023, APsA's Medical Student Education Committee (MSEC) established the Michael Balint Scholarship for Medical Students Interested in Psychoanalytic Work. It is named after a well-known psychoanalyst who introduced the concept of ‘patient-centredness’ into contemporary medicine. Dr Balint was also influential in setting up groups for physicians to discuss psychodynamic factors in their therapeutic relationships with patients. Known as Balint groups, they still retain relevance in modern patient care.^5^

The goal of this scholarship is to encourage psychodynamic and psychoanalytic thinking among medical students, which aligns with the mission and goals of the MSEC. The scholarship includes a $500 award, to be used for travel expenses to APsA's National Meeting, along with complimentary registration to the two main conferences hosted by APsA – the National Meeting (usually held in February) and the Annual Meeting (usually held virtually in June). The main criteria for applying include being actively enrolled in an accredited medical school at the time of the application. Along with their curriculum vitae, applicants are asked to submit a statement of interest expanding on their interest in psychodynamic/psychoanalytic concepts and how they would use the scholarship to advance this interest. In its first 2 years, the MSEC received robust responses – 25 applications in the inaugural year, followed by 31 applications the following year. The applications displayed diversity with respect to (self-identified) gender, sexual orientation, ethnicity, familiarity with psychodynamic principles and additional academic interests (although there seems to be a proclivity for interest in narrative medicine, creative arts in medicine and grassroots activism in medicine). A number of applicants attended the 2024 National Meeting (including some who had not received the scholarship) and they expressed excitement at what they learnt, including myths about psychoanalysis that were dispelled.

In conclusion, although newer evidence-based modalities of psychotherapy have proven to be effective and safe, psychodynamic psychotherapy and psychoanalysis still have much to offer, not just with respect to patient care but also in terms of professional development of medical trainees and practitioners. Although psychodynamic and psychoanalytic organisations are making strides to evolve and catch up with the contemporary zeitgeist, further efforts are required, including robust research on the effectiveness of this modality, along with devising innovative ways to recruit and retain interest in medicine. Hopefully, scholarships like those offered by APsA will help this cause.

## Declaration of interest

H.A. currently oversees applications for the Michael Balint Scholarship.
